# Peritoneal Tuberculosis in a Young Healthy Male Resembling Intra-Abdominal Malignancy

**DOI:** 10.7759/cureus.20677

**Published:** 2021-12-25

**Authors:** Rema F Alrashed, Alhanouf A Alkhuwaylidi, Khloud A Aldashash, Naif A Albati, Abdullah A Algarni, Helayel Almodhaiberi, Ibrahim Al Hasan

**Affiliations:** 1 Department of Surgery, Prince Sultan Military Medical City, Riyadh, SAU; 2 Department of Medicine, Princess Nourah Bint Abdulrahman University, Riyadh, SAU

**Keywords:** peritoneal tuberculosis (tb), abdominal pain, extra-pulmonary tuberculosis, intra-abdominal infection, tuberculosis

## Abstract

Intra-peritoneal tuberculosis is a rare disease, accounting for 1-2% of all tuberculosis cases. The absence of characteristic clinical picture of intra-peritoneal tuberculosis makes the diagnosis rarely easy for clinicians as it mimics malignancy. We present a case of a young male patient who presented with peritoneal tuberculosis, aiming to contribute to a better understanding of peritoneal tuberculosis in the literature.

## Introduction

Mycobacterium tuberculosis (TB) infection is one of the leading causes of death worldwide and continues to be a significant threat to global health. Extra-pulmonary manifestations are not common [1]. Extra-pulmonary TB (EPTB) may affect many body regions [1]. Moreover, peritoneal TB (PTB) is a less common entity of the disease with variable non-specific clinical, laboratory, and radiological features, making the diagnosis challenging and misleading to other diseases including malignancy [2,3]. We are presenting this case to contribute to a better understanding of PTB in the literature.

## Case presentation

A 21-year-old male presented to the general surgery clinic complaining of progressive generalized abdominal pain, more severe over the right upper quadrant, for six months. It was mild to moderate in intensity, aggravated by eating, and alleviated by oral analgesia ”paracetamol”. The pain was associated with constipation, abdominal distention, loss of appetite, and unintentional weight loss of 20 kg over five months. Moreover, it was associated with subjective fever, night sweats, and nausea without vomiting. The patient denied any medical or surgical history. The patient was living in Riyadh, Saudi Arabia, and was smoking three packets per day since the age of 18. He also reported a history of unprotected intercourse nine months prior to his presentation.

On physical examination, the patient was vitally stable, afebrile, conscious, alert, and oriented to place, person, and time. On abdominal examination, no scars or mass were inspected; however, there was tenderness on the right and left upper quadrants, but there was no rebound tenderness. Laboratory investigations were conducted upon admission in May 2021 (Table 1). Abdominal ultrasound showed mid-line soft tissue lesions likely arising from the pancreatic head or an enlarged pathological lymph node and free fluid. Computed tomography (CT) scan of the abdomen and pelvis with contrast was performed in May 2021, which showed a diffuse minimal thickening and enhancement of the peritoneum with omental nodularity with diffuse abdominopelvic ascites. Diffuse peritoneal wall thinking was noted as well as omental nodularity. Moreover, multiple enlarged necrotic abdominal lymph nodes were also noted, with the largest being celiac lymph node (2.5 cm). There were also small mildly enlarged lymph nodes in the left paraaortic region. There were also enlarged partially necrotic conglomerate lymph nodes in the lower mesentery, the largest measuring 2.4 cm. There was no significantly enlarged pelvic lymph node. The liver demonstrates homogeneous enhancement with a smooth outline. The spleen was mildly enlarged, measuring 13 cm without focal lesion. The pancreas, adrenal glands, and kidneys were unremarkable. The visualized lung bases demonstrate linear atelectatic changes. There was no pleural effusion. The impression of the CT scan was abdominal lymphadenopathy associated with ascites and peritoneal involvement. Chest CT with contrast was recommended and performed for the lung on May 9, 2021; the result showed small intrathoracic lymph nodes, the largest was at the subcarinal region and measured 1.5 cm. Subsegmental atelectasis was seen within the left upper lobe and right middle lobe. There were a few scattered tiny subpleural ground-glass changes in addition to minimal active small airway disease, with an impression of infectious or inflammatory bronchiolitis.

**Table 1:** Laboratory investigations upon admission

Labaratory investigation	Result
White blood count (WBC)	4.5 10^9^\L
Hemoglobin (Hbg)	11.7 g/L
Platelets	117 10^9^\L
International normalized ratio (INR)	1
Alanine transferase (ALT)	11 unit/L
Alkaline phosphatase (ALP)	58 unit/L
Blood urea nitrogen (BUN)	2.2 mole/L
Creatinine (Cr)	6 mole/L
Sodium (Na)	136 mole/L
Potassium (K)	3.9 mole/L
Total bilirubin (T.Bili)	12 mcmol/L
Quantiferon	+ve

A request to the interventional radiology department was sent to take a biopsy, which was obtained from the peritoneal thickening and yielded no conclusive results (acid-fast bacillus test was performed for the specimen taken and was negative). Lymph node biopsy was not feasible due to the proximity of the affected lymph nodes to the aorta and celiac plexus. The decision to take the patient for surgery was made, and the patient was consented for laparoscopic exploration with possible need for open surgery and excision lymph node biopsy.

**Figure 1:**
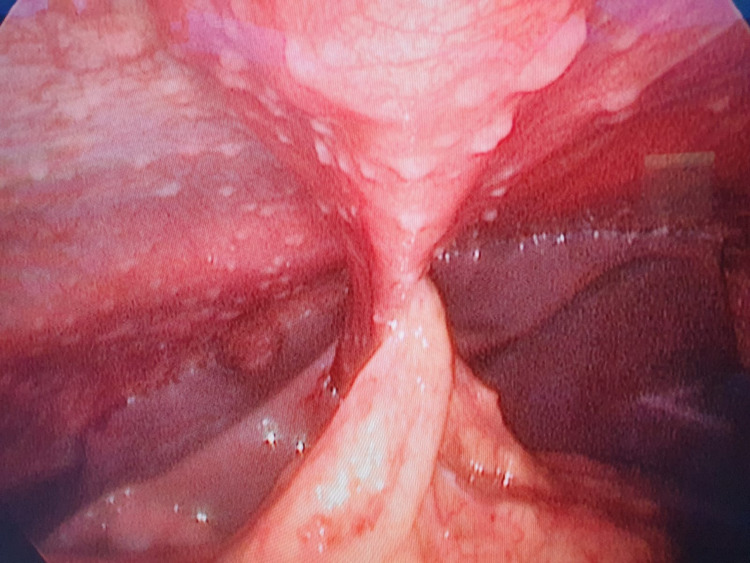
Laparoscopic view of diffuse intra-abdominal deposits

Laparoscopic examination under general anesthesia revealed serous fluid over the four abdominal quadrants, from which a sample was taken for culture and analysis. Furthermore, there was a notably large number of peritoneal nodules (Figure 1) from which excisional biopsy was taken for histopathology. In the postoperative period, the patient was well with no complaints; however, he spiked low-grade fever (38.4 degrees Celsius) twice, for which discharge was held until an urgent consultation from infectious diseases (ID) service was obtained, The ID examined the patient and suggested to send three specimens of sputum for polymerase chain reaction for TB, which were negative. The patient was then discharged home in a good condition with follow-up with the ID clinic and general surgery clinic.

Histopathological examination of the excisional biopsy specimen revealed nodular fragments of fibrofatty and skeletal muscle tissue, with clusters of epithelioid histiocytes, admixed lymphocytes, and neutrophils with central necrosis. Scattered multinucleated Langhans-type giant cells were seen. Acid-fast bacillus test was performed, which was positive for the excisional biopsy specimen.

## Discussion

Mycobacterium TB is a pathogenic bacteria whose infection accounts for a significant morbidity and0020mortality. EPTB is uncommon where it occurs in around 12% of patients; however, its incidence in Arabia can reach up to 30% [1,4]. The risk factors for EPTB include HIV status, which remained strongly associated with EPTB, and it is also found to be more in female patients as well as non-Hispanic black race.

EPTB may affect bones and/or joints, genitourinary tract, and cervical and peritoneal lymphatics [2].

Moreover, PTB is a less common entity of the disease with an incidence of 4.7%, and with variable non-specific clinical, laboratory, and radiological features, making the diagnosis challenging and misleading to other diseases including malignancy [2,3]. PTB has non-specific clinical manifestations, which consist of fatigue, fever, loss of weight and appetite, vague abdominal pain, ascites, and palpable abdominal masses. This makes it challenging to differentiate between PTB and malignancy, especially that it can be associated with raise in cancer antigen 125 (CA-125) [5]. Ascitic fluid tapping and analysis for acid-fast bacilli is of a less diagnostic value compared to Quantiferon. However, new molecular tests for ascitic fluid, including GeneXpert MTB/RIF and GeneXpert MTB/RIF Ultra, interferon gamma, and adenosine deaminase, have a sensitivity and a specificity higher than 90% and are recommended by the World Health Organization (WHO). Furthermore, radiological abdominal investigations do not provide much help in distinguishing between peritoneal carcinomatosis and PTB, as both will show irregular peritoneal nodularity and ascites [5]. Our case had a presentation similar to peritoneal carcinomatosis. Although in the literature, PTB is associated with low immune status and other comorbidities, our patient is a young medically free patient with good functional status.

Since the radiological and laboratory studies are not sensitive and specific in PTB, the diagnosis is frequently made by peritoneal biopsy and histological analysis. This can be established by diagnostic laparoscopy; however, caution should be practiced as conversion to laparotomy can reach up to 10% as severe adhesions can be encountered. Laparoscopy helps in achieving an early diagnosis of PTB and starting the medical management without further delay [6].

## Conclusions

PTB has similar characteristics to peritoneal carcinomatosis, which makes the diagnosis often challenging for physicians. An early and proper diagnosis of PTB helps in initiating medical management quickly and helps in reducing morbidity and mortality.
